# Relationship Between Social Capital and Depressive Symptoms Among Type 2 Diabetes Mellitus Patients in Northwest China: A Mediating Role of Sleep Quality

**DOI:** 10.3389/fpsyt.2021.725197

**Published:** 2021-09-20

**Authors:** Liqun Wang, Jiangping Li, Yuqi Dang, Haiyu Ma, Yang Niu

**Affiliations:** ^1^Department of Epidemiology and Statistics, School of Public Health and Management, Ningxia Medical University, Yinchuan, China; ^2^Key Laboratory of Environmental Factors and Chronic Disease Control, Ningxia Medical University, Yinchuan, China; ^3^Department of Endocrinology, Yinchuan Hospital of Traditional Chinese Medicine, Yinchuan, China; ^4^School of Traditional Chinese Medicine, Ningxia Medical University, Yinchuan, China; ^5^Key Laboratory of the Ningxia Ethnomedicine Modernization, Ministry of Education, Ningxia Medical University, Yinchuan, China

**Keywords:** social capital, sleep quality, depressive symptoms, mediation, T2DM patients

## Abstract

**Objective:** There are few studies about the relationship between social capital (SC) and depression among type 2 diabetes mellitus (T2DM) patients, and the mechanism explaining how SC leads to decreased depression is unclear. The current study aims to explore the relationship between SC and depressive symptoms among the T2DM patients in northwest China, with a particular focus on the mediating role of sleep quality.

**Methods:** A cross-sectional study of 1,761 T2DM patients from Ningxia Province was conducted. The Center for Epidemiological Survey Depression Scale (CES-D) and self-report sleep quality questionnaire coupled with the SC scales were administered during the face-to-face survey. The Bootstrap methods PROCESS program is employed to test the mediation model.

**Results:** The prevalence of depressive symptoms among T2DM patients was 24.8%. After controlling for covariates, the SC (*r* = −0.23, *p* < 0.001) was negatively correlated with CES-D score; the sleep quality was also negatively correlated with CES-D score (*r* = −0.31, *p* < 0.001); and the SC was positively correlated with sleep quality (*r* = 0.10, *p* < 0.001). Logistic regression analysis showed that SC was inversely related to the risk of depressive symptoms. Meanwhile, sleep quality was negatively associated with depressive symptoms. Sleep quality has mediated the relationship between SC and depressive symptoms among T2DM patients (explaining 12.6% of the total variance).

**Conclusions:** We elucidated how SC interacted with depressive symptoms through the mediation pathway of sleep quality using a representative sample of the Chinese diabetes patients. The findings indicate that the improvement of SC and sleep quality may help in maintaining mental health among T2DM patients. Hence, clinicians can suggest that patients communicate more with others to improve the SC and, in turn, maintain their health.

## Introduction

As one of the most common chronic non-communicable diseases in the world, diabetes mellitus has become an increasingly serious global health problem (1). More than 90% of diabetes mellitus cases are classified as type 2 diabetes mellitus (T2DM) (2). China has the largest diabetes patients in the world; the number of diabetes patients in China reached 114.4 million in 2017 (3). Prolonged high blood sugar of T2DM can cause retinopathy, peripheral neuropathy, and diabetic nephropathy and also increase the risk of complications, such as cerebrovascular disease, peripheral vascular disease, and stroke (4), further causing patient's poor life quality and premature death (5, 6).

Apart from physical impairments, T2DM is also inversely related to mental health. Several studies have found that depression or depressive symptoms were increased in diabetes patients (7–10). In particular, depression occurrence was two to three times higher in individuals with diabetes mellitus (11). Depression has a synergistic effect in patients with diabetes mellitus, thereby reducing the quality of life (12) and increasing the risk for complications and mortality (13). Therefore, it is critical to identify factors influencing depressive symptoms in diabetes patients, and increased awareness for depression in diabetes might improve the effectiveness of treatment. Sleep quality is significantly associated with depression. A previous study reported that poor perceived sleep quality was associated with a greater level of depression in a Korean elderly population (14). There was a significant correlation between increased depressive symptoms and subjective sleep quality among the cardiac inpatients (15). A meta-analysis revealed that lack of good sleep quality in an old individual is significantly related with depression (16). Moreover, sleep quality is an important mediator of the relationship between perceived stress and depression (17).

Social capital (SC) is a characteristic of social life, including interpersonal trust, norms of reciprocity, mutual aid, and social involvement (like socializing with friends, relatives, colleagues, or neighbors) (18), which is linked with several beneficial health outcomes (19, 20). As a social determinant of health, SC may play an essential role in protecting individuals from depression. A recent study has found that older people with higher SC had a smaller chance to develop depression (21). Specifically, the study found an inverse association between SC and depression among migrant hypertensive patients (22). Besides, SC was positively related to sleep quality (23). A notable example is one study that revealed neighborhood social cohesion was significantly positively related with sleep quality (24).

There are still few studies that examined the relationship between SC and depressive symptoms among diabetes patients, and few empirical studies have explored the underlying mechanisms through which SC affects depressive symptoms. To fill this gap, this study aims to explore the relationship between SC and depressive symptoms among the diabetes patients in northwest China, with a particular focus on the mediating role of sleep quality. This study could shed light on future studies concerning the impact of SC on depressive symptoms among the diabetes patients and promote the effectiveness of treatment.

**Figure 1:**
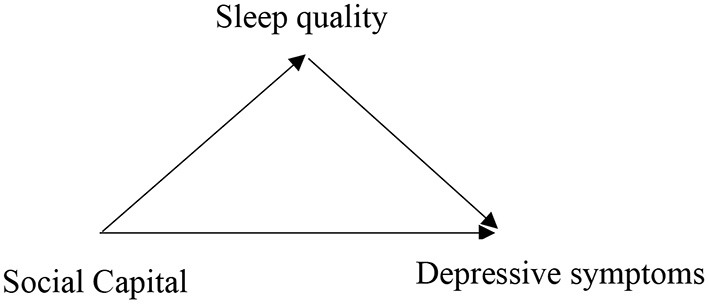
Diagram for the mediation analysis.

Taking into account the above concern, the current study began with the following hypotheses: Hypothesis 1: SC will be negatively and directly related to depressive symptoms; Hypothesis 2: SC will be positively and directly related to good sleep quality; Hypothesis 3: poor sleep quality will be positively and directly related to depressive symptoms; and Hypothesis 4: SC will be positively and indirectly related to depressive symptoms *via* sleep quality (as shown in Figure 1).

## Materials and Methods

### Subjects and Procedure

Data were abstracted from a cross-sectional survey conducted from August 2019 to November 2020 at Ningxia Province, China. The participants were selected using a probability proportionate to size (PPS) sampling method; the detailed sampling process was as follows: in the first stage, the primary sampling units (PSU) were selected from all 48 government hospitals (as recorded in the Ningxia Statistics Bureau). In the second stage, all the hospitals were stratified by hospital types as Chinese medicine hospitals and general hospitals. In the final sampling stage, given the hospital size and number of beds, 10 hospitals (5 Chinese medicine hospitals and 5 general hospitals) were selected using PPS sampling methods. Then, data of type 2 diabetes patients were collected in the department of endocrinology of each hospital. Ultimately, 1,761 participants were included in this study. The inclusion criteria were: (a) living at the present address for at least 6 months and (b) aged 18 years or older. The exclusion criteria were the following: (a) with severe mental disorders; (b) a severe illness that prevents communication; (c) deafness, aphasia, or other language barriers; (d) pregnancy or lactation; (e) diabetic ketoacidosis in the past month; (f) with malignant tumor; and (g) refuse to sign the informed consent. The Institutional Review Board of the Ningxia Medical University approved this study. All the participants provided a written consent form at the beginning of the survey.

### The Field Process

Participants will be recruited in the department of endocrinology of each hospital by Chinese medicine doctors because part of the study content is related to Chinese medicine syndrome. The trained Chinese medicine doctors served as investigators. The doctors filter the participants *via* the Hospital Information System (HIS) according to the inclusion and exclusion criteria. With the hospital leaders' cooperation, the investigators visited the patients to guide them to finish the survey and then described our study and questionnaire. Under the patients' agreement, our investigators read the questions one by one to them and then recorded their answers, and the survey lasted ~30 min. The finished questionnaire was double-checked immediately by a separate supervisor in the field.

### Depressive Symptoms

The Center for Epidemiological Survey Depression Scale (CES-D) was employed to assess the depressive symptoms. CES-D was conducted by Radloff in 1977; it was mainly used for epidemiological investigations to screen out subjects with depressive symptoms and also used as a clinical examination to assess the severity of depressive symptoms. The scale contains 20 items, and 4 of them are reverse scoring (25). The total score ranged from 0 to 60, with higher scores indicating higher levels of depression, and a cut-off value of 16 was recommended to the study (26). The scale has high reliability and validity among the Chinese population (26).

### Social Capital

SC was evaluated using the SC scale to cover the two dimensions of SC (social cohesion and social interaction) developed by Mujahid (27). The social cohesion subscale consists of four items: (1) People around here are willing to help their neighbors, (2) People in my neighborhood generally get along with each other, (3) People in my neighborhood can be trusted, and (4) People in my neighborhood share the same values. Each item ranged from 1 to 5 (1 = strongly disagree, 2 = disagree, 3 = neutral, 4 = agree, and 5 = strongly agree). Cronbach's alpha was 0.88 among the Chinese sample (28) and 0.95 among this sample. The social interaction scale consists of five items: (1) You and other people in the community (village) help each other (e.g., look after children, help buy something, and borrow tools), (2) When a neighbor is not at home or going out, you can help him look after the house or property, (3) People in the community (village) talk about each other's personal matters (children care, exercise, etc.), (4) Participate in group activities together with people in the community (village), and (5) Communicate with each other on the street. Each item was scored from 1 to 4 in response to a 4-point Likert scale (from never to often). A previous study has shown that the Chinese version of the social interaction scale has good reliability and validity (28). The Cronbach's alpha in this sample was 0.92.

### Sleep Quality

Sleep quality was self-reported *via* the question “What do you think of your sleep quality?,” with answers poor, little good, and good.

### Covariates

The covariates were classified into socio-demographic variables and health variables. Socio-demographic information including age, gender (male vs. female), ethnicity (Han vs. minority), residence (urban vs. rural), educational attainment, marital status (unmarried, married, widowed, or divorced), occupation (farmer vs. others), and family income (as measured by the self-reported family average individual income per month and was divided into five groups: <1,000 RMB, 1,000–1,999 RMB, 2,000–2,999 RMB, 3,000–4,999 RMB, and 5,000 RMB or more) was collected using a standard form.

Health variables were described as follows. The data about body mass index [BMI = weight (kg)/height (m^2^)], smoking (defined as at least one cigarette per day and last for 6 months or more), and alcohol use (defined as a drink at least one glass of alcohol, which equals to 1/2 bottle of beer or 125-milliliter grape wine or fruit wine or 40-milliliter white wine, in the past 12 months) were collected. Tea drinking frequency was assessed by asking the question “How often do you drink tea (days per week)?,” with possible responses: once a day or more, 5–6 times/week, 3–4 times/week, 1–2 times/week, less than once a week, and never. Exercise was assessed by asking the question “Do you perform at least 30 min of physical activity at work and/or leisure time more than 4 days a week?,” with a yes/no response. Afternoon nap (yes vs. no), sleep duration (continuous data), other chronic diseases (yes vs. no), T2DM complications (yes vs. no), and disease duration (continuous data) were abstracted from the medical records.

### Statistical Analyses

Analyses were performed using the Statistical Package for the Social Sciences (SPSS) version 24.0 (SPSS Inc., Chicago, IL, USA). Means and standard deviations were used to describe continuous variables; counts and proportions were used to describe categorical variables. A correlation matrix was created using partial correlations after controlling for age, gender, ethnicity, marital status, education, occupation, economic condition, residence, smoking, alcohol use, tea drinking, physical exercise, BMI, other chronic diseases, T2DM complications, disease duration, afternoon nap, and sleep duration. A logistic regression model was used to examine the association of SC with sleep quality and depressive symptoms. Bootstrap methods of PROCESS developed by Hayes (29) were employed to test the mediation effect of sleep quality on the relationship between SC and depressive symptoms. The bias-corrected percentile bootstrap confidence interval does not contain 0 to indicate that the mediation effect is statistically significant (30).

## Results

### Demographic Characteristics of Participants

The demographic characteristics of the participants are shown in Table 1. The average age was 66.9 [standard deviation (SD) =5.3] years, with a range of 21–85 years. Slightly over half (54.8%) were male, approximately one fifth was illiterate, and ~93.2% were married. The mean sleep duration was 8.2 (SD = 1.2) h, and the mean score of SC was 27.7 (SD = 2.1). The mean score of CES-D was 11.1 (SD = 7.3), and the prevalence of depression symptoms was 24.8%. Furthermore, 394 (22.4%) participants reported poor sleep quality.

**Table 1:** Demographic characteristics of the participant.

	**Total *n* = 1,761**	**CES-D score ≥ 16*n* = 437**	**CES-D score <16^#^ *N* = 1,324**	***p*-value**
Age, mean (SD), years	58.6 (12.2)	60.9 (11.7)	57.8 (12.2)	<0.001
Gender, male, *n* (%)	965 (54.8)	193 (44.2)	772 (58.3)	<0.001
Ethnicity, Han, *n* (%)	1,288 (73.2)	307 (70.3)	981 (74.1)	0.116
Residence, urban, *n* (%)	1,102 (62.6)	246 (56.3)	856 (64.7)	0.002
Marital status, married, *n* (%)	1,461 (93.2)	390 (89.2)	1,251 (94.5)	<0.001
Education level, *n* (%)				0.001
Illiterate	351 (19.9)	100 (22.9)	251 (19.0)	
Primary	394 (22.4)	121 (27.7)	273 (20.6)	
Junior and senior	621 (35.3)	131 (30.0)	490 (37.0)	
College degree or above	395 (22.4)	85 (19.5)	310 (23.4)	
Occupation, farmer, *n* (%)	709 (40.3)	214 (49.0)	495 (37.4)	<0.001
FPCMI, <2,000, *n* (%)	685 (38.9)	193 (44.2)	492 (37.2)	0.022
Smoking, yes, *n* (%)	396 (22.5)	84 (19.2)	312 (23.6)	0.059
Alcohol use, yes, *n* (%)	368 (20.9)	62 (14.2)	306 (23.1)	<0.001
Drinking tea, *n* (%)				0.014
Every day	497 (28.2)	116 (26.5)	381 (28.8)	
5–6 days a week	54 (3.1)	13 (3.0)	41 (3.1)	
3–4 days a week	127 (7.2)	23 (5.3)	104 (7.9)	
1–2 days a week	60 (3.4)	7 (1.6)	53 (4.0)	
Occasion	370 (21.0)	110 (25.2)	260 (19.6)	
Never	653 (37.1)	168 (38.4)	485 (36.6)	
Physical exercise, yes, *n* (%)	1,175 (66.7)	263 (60.2)	912 (68.9)	0.001
BMI, mean (SD), kg/m^2^	25.0 (14.2)	24.4 (3.4)	25.2 (6.5)	0.022
Other chronic diseases, yes, *n* (%)	1,160 (65.9)	311 (71.2)	849 (64.1)	0.007
T2DM complications, yes, *n* (%)	1,059 (60.1)	288 (65.9)	771 (58.2)	0.005
Disease duration, mean (SD), years	8.4 (7.6)	9.2 (7.6)	8.1 (7.5)	0.008
Afternoon nap, yes, *n* (%)	866 (49.2)	203 (46.5)	663 (50.1)	0.189
Sleep duration, mean (SD), h	8.2 (1.2)	8.3 (1.1)	8.2 (1.2)	0.012
Sleep quality, poor, *n* (%)	394 (22.4)	167 (38.2)	227 (17.1)	<0.001
CES-D score, mean (SD)	11.1 (7.3)	21.3 (4.9)	7.8 (4.3)	<0.001
SC score, mean (SD)	27.7 (2.1)	26.8 (6.5)	28.0 (6.5)	<0.001

*FPCMI, family per capita monthly income; SD, standard deviation; BMI, body mass index; T2DM, type 2 diabetes mellitus; CES-D, Center for Epidemiological Survey Depression Scale; SC, social capital*.

# *Compared with CES-D score ≤ 16*.

### The Binary Correlation Matrix

The partial correlation matrix is shown in Table 2. After controlling for socio-demographic variables (age, gender, ethnicity, marital status, education, occupation, economic condition, residency) and health variables (smoking, alcohol use, tea drinking, physical exercise, BMI, other chronic diseases, T2DM complications, disease duration, afternoon nap, sleep duration), the SC (*r* = −0.23, *p* < 0.001) was negatively correlated with CES-D score; the sleep quality was also negatively correlated with CES-D score (*r* = −0.31, *p* < 0.001); and the SC was positively correlated with sleep quality (*r* = 0.10, *p* < 0.001).

**Table 2:** Correlation matrix (*n* = 1,761).

	**Mean**	**SD**	**CES-D**	**SC**	**Sleep quality**
CES-D	11.1	7.3	1		
SC	27.7	6.5	−0.23^**^	1	
Sleep quality	2.1	0.7	−0.31^**^	0.10^**^	1

** *p <0.001*.

^*^p <0.05.

*SD, standard deviation*.

*After controlling for age, gender, ethnicity, marital status, education, occupation, economic condition, residence, smoking, alcohol use, tea drinking, physical exercise, BMI, other chronic diseases, T2DM complications, disease duration, afternoon nap, and sleep duration*.

### Categorical Analyses

As Table 3 shows, categorical analyses revealed that SC was inversely related to risk of depressive symptoms. Meanwhile, sleep quality was negatively associated with depressive symptoms (**model 1**). After controlling for covariates (age, gender, ethnicity, marital status, education, occupation, economic condition, residency, smoking, alcohol use, tea drinking, physical exercise, BMI, other chronic diseases, T2DM complications, course of disease, afternoon nap, sleep duration), these findings persisted significantly (**model 2**). In **model 3**, the interaction between SC and sleep quality did not have statistical significance, indicating that there was no moderating relationship between SC and sleep quality and possible mediation exists.

**Table 3:** Logistic regression model for interaction between SC and sleep quality on depressive symptoms (*n* = 1,761).

**Variables**	**Model 1**		**Model 2**		**Model 3**	
	***p*-value**	**OR (95% CI)**	***p*-value**	**OR (95% CI)**	***p*-value**	**OR (95% CI)**
SC (reference = low)	0.001	0.97 (0.95, 0.99)	<0.001	0.96 (0.94, 0.98)	0.001	0.92 (0.87, 0.97)
Sleep quality (reference = poor)	<0.001	0.43 (0.37, 0.51)	<0.001	0.44 (0.37, 0.53)	<0.001	0.23 (0.12, 0.47)
Age	NA	NA	0.128	1.11 (0.97, 1.27)	0.156	1.10 (0.96, 1.26)
Gender (reference = female)	NA	NA	0.010	0.68 (0.51, 0.91)	0.009	0.68 (0.50, 0.91)
Ethnicity (reference = minority)	NA	NA	0.127	0.81 (0.62, 1.06)	0.146	0.82 (0.62, 1.07)
Residence (reference = rural)	NA	NA	0.046	0.72 (0.52, 0.99)	0.053	0.73 (0.53, 1.00)
Marital status (reference = married)	NA	NA	0.019	0.60 (0.39, 0.92)	0.018	0.60 (0.39, 0.92)
Education (reference = illiterate)	NA	NA	0.032	1.20 (1.02, 1.42)	0.037	1.20 (1.01, 1.41)
Occupation (reference = non-farmers)	NA	NA	0.012	1.58 (1.11, 2.25)	0.013	1.57 (1.10, 2.24)
FPCMI	NA	NA	0.311	0.94 (0.85, 1.05)	0.274	0.94 (0.84, 1.05)
Smoking (reference = no)	NA	NA	0.301	1.21 (0.84, 1.72)	0.305	1.21 (0.84, 1.73)
Alcohol use (reference = no)	NA	NA	0.154	0.76 (0.52, 1.11)	0.195	0.78 (0.53, 1.14)
Drinking tea (reference = no)	NA	NA	0.095	0.95 (0.89, 1.01)	0.095	0.95 (0.89, 1.01)
Physical exercise (reference = no)	NA	NA	0.001	0.67 (0.52, 0.86)	0.002	0.67 (0.53, 0.86)
BMI	NA	NA	0.009	0.80 (0.68, 0.95)	0.009	0.80 (0.68, 0.95)
Other chronic diseases (reference = no)	NA	NA	0.425	1.12 (0.85, 1.46)	0.389	1.13 (0.86, 1.47)
T2DM complications (reference = no)	NA	NA	0.185	1.19 (0.92, 1.55)	0.192	1.19 (0.92, 1.55)
Course of disease	NA	NA	0.157	1.09 (0.97, 1.23)	0.155	1.09 (0.97, 1.23)
Afternoon nap (reference = no)	NA	NA	0.663	1.06 (0.83, 1.34)	0.661	1.06 (0.83, 1.34)
Sleep duration	NA	NA	0.097	1.09 (0.98, 1.21)	0.106	1.09 (0.98, 1.21)
SC × sleep duration	NA	NA	NA	NA	0.302	1.01 (0.99, 1.04)

*Model l = SC or sleep quality separately; model 2 = model l + covariates (age, gender, ethnicity, marital status, education, occupation, economic condition, residence, smoking, alcohol use, tea drinking, physical exercise, BMI, other chronic diseases, T2DM complications, disease duration, afternoon nap, sleep duration); model 3 = model 2 + interaction between SC and sleep quality; SC, social capital; OR, odds ratio; 95% CI, 95% confidence interval; NA, not applicable*.

### Mediation Effect of Sleep Quality on the Relationship of SC and Depression Symptoms Among T2DM Patients

As shown in Table 4, after controlling for covariates, there is a significant mediation effect of sleep quality on the relationship between SC and depressive symptoms. The results showed that both the direct effect (*p* < 0.001) and the indirect effect (*p* < 0.001) were significant. The mediation effect explained 12.6% (−0.033/−0.262) of the total variance.

**Table 4:** The mediating effect of sleep quality on the relationship between SC and depressive symptoms^*^.

**Effect**				**Bias-corrected 95% CI**	
	**β**	***SE***	***p*-value**	**Lower**	**Upper**
Total effect	−0.262	0.027	<0.001	−0.316	−0.219
Indirect effects	−0.033	0.001	<0.001	−0.051	−0.017
Direct effects	−0.229	0.026	<0.001	−0.281	−0.178

* *After controlling for age, gender, ethnicity, marital status, education, occupation, economic condition, residence, smoking, alcohol use, tea drinking, physical exercise, BMI, suffer from other disease, T2DM complications, disease duration, afternoon nap, and sleep duration*.

## Discussion

To the best of our knowledge, this was the first study to investigate the relationship between SC and depressive symptoms among type 2 diabetes patients in China. As hypothesized, we found: (1) depressive symptoms were prevalent among T2DM patients in northwest China; (2) SC was negatively related to depressive symptoms and positively associated with sleep quality; poor sleep quality was positively related to depressive symptoms; and (3) in the relationship between SC and depressive symptoms, sleep quality mediated the association, the mediation effect accounting for 12.6% of the total effect.

In this study, our finding of 24.8% T2DM patients with depressive symptoms (CES-D score ≥ 16) is lower than the prevalence of depressive symptoms among Japanese (29.9%) (31) and Nepalese (44.1%) (32) T2DM patients and higher than in northeast China (8.76%) (33). The reasons for this phenomenon may be owing to different samples, survey regions, and measurement instruments or clinical factors, such as disease duration and severity (2). The incidence of depression is higher in diabetic patients than in the general population (34); this may be due to poor sleep quality (35), long-term medication, or several complications (36). In addition, many studies suggested that altered hypothalamic–pituitary–adrenal (HPA) axis (37), microvascular hypothesis (38), insulin resistance (39), and cytokine-mediated inflammatory response (40) were the possible mechanisms of the association between T2DM and depression. Based on this, more attention should be given to T2DM patients concerning depression intervention.

In line with the previous findings, we found that SC was negatively associated with depressive symptoms among T2DM samples. A previous study among T2DM patients showed similar findings that social support had a significant decremental association with depression that could contribute to improved health outcomes (41). Another study among the hypertension patients also showed that higher SC reduced the chance of developing depression (22). The possible mechanisms were that SC plays a crucial role in the psychosocial adjustment of the patient toward chronic disease, even in periods of depression, and SC can work therapeutically in short or long term (42). In addition, participants engaged with their partners and shared experiences and practices in coping with their conditions (41). Hence, patients with chronic diseases need more support, trust, and social interaction.

It difficult to infer any causal pathways between sleep and depression. However, quite a few longitudinal studies (43, 44) have consistently highlighted the role of sleep disorder in the prediction of depression. Our results showed that sleep quality was negatively associated with CED-S score among T2DM patients. Poor sleep quality might be a risk factor for depression, consistent with prior research that psychiatric disorders, such as depression, may be negatively related to quality of sleep (45). The study suggested that sleep quality may initiate and/or exacerbate mood disturbance, and that it may be possible to improve sleep quality to maintain mood (46).

Compared with higher SC, poor sleep quality occurred in those who have lower SC. This finding was consistent with a previous study that found lower neighborhood social cohesion was negatively associated with sleep quality among American adults (24). The possible mechanism may be that a lower SC may cause dysregulation of the HPA axis, which mediates responses to stressors and is known to result in sleep disorders, such as insomnia and poor sleep quality (47). Moreover, our mediation analysis results suggest that sleep quality mediated the relationship between SC and depressive symptoms. Namely, SC partly affected depressive symptoms by sleep quality among the T2DM patients. Besides, except for sleep quality, there are other possible mechanisms between SC and depression, like social engagement (48), loneliness (49), life satisfaction (50), and traumatic life experience (51), etc.

Given the increasing rate of depression among T2DM patients, the present findings have relevance for understanding the mechanisms of how SC is linked with depressive symptoms and provide primary evidence for developing clinical interventional program for depression among T2DM patients. Several limitations were identified. First, only correlation rather than causal relationship due to the cross-sectional design and prevents making causal inferences from the relationships between SC and depressive symptoms reported here. Hence, further longitudinal design would be necessary to determine causal relationships in the future. Second, other potential mediators of the relationship between SC and depression, such as cognitive function, were not assessed. Third, due to the feasibility consideration, sleep quality was collected *via* a self-reported survey question; it may involve recall bias even though it has been found to have a reasonable correlation with actigraphic measurement (52).

## Conclusion

In summary, this study reveals that SC has a positive effect on depressive symptoms among the T2DM patients. Furthermore, this study provides new evidence indicating that sleep quality partly mediates the relationship between SC and depressive symptoms, and that SC and sleep quality may require attention to reduce depressive risks from diabetes mellitus. Hence, clinicians can suggest that patients communicate more with others to improve their SC and, in turn, maintain their health. Further prospective study is needed to confirm this finding due to the cross-sectional design.

## Data Availability Statement

The original contributions generated for the study are included in the article/supplementary material, further inquiries can be directed to the corresponding author/s.

## Ethics Statement

The studies involving human participants were reviewed and approved by the Institutional Review Board of the Ningxia Medical University. The patients/participants provided their written informed consent to participate in this study.

## Author Contributions

LW, JL, and YN designed the study. LW, YD, and HM collected the data. LW and JL analyzed the data. LW and YN wrote the article. JL and YN reviewed the article. All authors contributed to the article and approved the submitted version.

## Funding

This work was supported by the research on hygienic appropriate technology of primary medical institutions in minority region (2013BAI05B01).

## Conflict of Interest

The authors declare that the research was conducted in the absence of any commercial or financial relationships that could be construed as a potential conflict of interest.

## Publisher's Note

All claims expressed in this article are solely those of the authors and do not necessarily represent those of their affiliated organizations, or those of the publisher, the editors and the reviewers. Any product that may be evaluated in this article, or claim that may be made by its manufacturer, is not guaranteed or endorsed by the publisher.
